# Accuracy of parasitological and immunological tests for the screening of human schistosomiasis in immigrants and refugees from African countries: An approach with Latent Class Analysis

**DOI:** 10.1371/journal.pntd.0005593

**Published:** 2017-06-05

**Authors:** Anna Beltrame, Massimo Guerriero, Andrea Angheben, Federico Gobbi, Ana Requena-Mendez, Lorenzo Zammarchi, Fabio Formenti, Francesca Perandin, Dora Buonfrate, Zeno Bisoffi

**Affiliations:** 1 Centre for Tropical Diseases, Ospedale Sacro Cuore Don Calabria, Negrar, Verona, Italy; 2 University of Verona, Department of Computer Science, Verona, Italy; 3 Barcelona Institute for Global Health, ISGlobal-CRESIB, Universitat de Barcelona, Barcelona, Spain; 4 Infectious and Tropical Diseases Department, Azienda Ospedaliera Universitaria Careggi, Florence, Italy; Swiss Tropical and Public Health Institute, SWITZERLAND

## Abstract

**Background:**

Schistosomiasis is a neglected infection affecting millions of people, mostly living in sub-Saharan Africa. Morbidity and mortality due to chronic infection are relevant, although schistosomiasis is often clinically silent. Different diagnostic tests have been implemented in order to improve screening and diagnosis, that traditionally rely on parasitological tests with low sensitivity. Aim of this study was to evaluate the accuracy of different tests for the screening of schistosomiasis in African migrants, in a non endemic setting.

**Methodology/Principal findings:**

A retrospective study was conducted on 373 patients screened at the Centre for Tropical Diseases (CTD) in Negrar, Verona, Italy. Biological samples were tested with: stool/urine microscopy, Circulating Cathodic Antigen (CCA) dipstick test, ELISA, Western blot, immune-chromatographic test (ICT). Test accuracy and predictive values of the immunological tests were assessed primarily on the basis of the results of microscopy (primary reference standard): ICT and WB resulted the test with highest sensitivity (94% and 92%, respectively), with a high NPV (98%). CCA showed the highest specificity (93%), but low sensitivity (48%). The analysis was conducted also using a composite reference standard, CRS (patients classified as infected in case of positive microscopy and/or at least 2 concordant positive immunological tests) and Latent Class Analysis (LCA). The latter two models demonstrated excellent agreement (Cohen’s kappa: 0.92) for the classification of the results. In fact, they both confirmed ICT as the test with the highest sensitivity (96%) and NPV (97%), moreover PPV was reasonably good (78% and 72% according to CRS and LCA, respectively). ELISA resulted the most specific immunological test (over 99%). The ICT appears to be a suitable screening test, even when used alone.

**Conclusions:**

The rapid test ICT was the most sensitive test, with the potential of being used as a single screening test for African migrants.

## Author summary

Schistosomiasis is probably the most important of the neglected tropical diseases (NTD) caused by helminthes (worms). It is acquired bathing in freshwater in endemic areas. The life cycle is complex and involves freshwater snails. Schistosomiasis, caused by *Schistosoma mansoni*, *S*. *haematobium* and less frequently by other species, affects more than 200 million people, mostly in Africa, and may chronically cause irreversible damage of the liver (*S*. *mansoni*) or of the kidneys and the urinary tract, including cancer of the bladder (*S*. *haematobium*). As in chronic infections eggs of both species are often missed by microscopy of faeces and urine, with this retrospective study we evaluate the accuracy of different, alternative diagnostic tests, for the screening of schistosomiasis in African migrants and asylum seekers, of whom many thousands reach the Italian coast every year proceding from the most endemic areas. The most interesting finding of our study is that a rapid diagnostic test for antibody detection in blood, easy to use as a point-of-care tool, resulted the most sensitive of the five tests evaluated, and thus is very promising as a screening tool even when used without any additional test.

## Introduction

Schistosomiasis is a fluke worm infection affecting at least 78 countries and more than 200 million people worldwide, and causing an estimated burden of 3.3 million disability-adjusted life-years (DALYs) [1,2].

Ninety-three percent of the global cases occur in sub-Saharan Africa, mostly caused by the species *Schistosoma mansoni* and *S*. *haematobium*. In this area, approximately 300,000 deaths due to schistosomiasis are estimated annually [3–6]. A huge wave of migrants, in particular asylum seekers, has reached Italy from sub-Saharan Africa in the past few years [7]. The health screening of newly arrived migrants is usually limited to detect potentially transmissible diseases such as tuberculosis and scabies. Schistosomiasis, often clinically silent, is not routinely screened. Given the severe complications related to both *S*. *mansoni* and *S*. *haematobium* [8–12], and the availability of an effective and relatively inexpensive treatment [1,13,14], adequate protocols for screening and treatment of migrants are required. Further, the recent outbreak of urinary schistosomiasis in Corsica [15], originated by a West African strain [16,17], is a useful reminder that local transmission of schistosomiasis in Europe is still possible [18,19].

The estimated prevalence of schistosomiasis in African migrants, reported from a scarce European literature, ranged from 9–15% if based on use of microscopy [20,21] to 5.8–24.7% if based on ELISA serology [22,23]. Clearly, all data obtained from reference centers are to be taken with caution as they may not be representative of the general population. These prevalence estimates are based on relatively insensitive tests, which under represent infections with low parasite loads. Traditionally, the gold standard for the diagnosis of schistosomiasis has been considered to be the urine and stool microscopy of several specimens [24]. Microscopy is 100% specific, but the sensitivity (40–60%) varies with the intensity of infection, the number of specimens collected, and the circadian and day-to-day variation of egg counts in stool and/or urine [25–27]. The diagnosis of schistosomiasis by detection of specific antibodies (with tests generally based on crude antigens of *S*. *mansoni*) is more sensitive than microscopy, particularly in light infections [26,28]. However, commercial serologic tests for schistosomiasis have a sub-optimal sensitivity too, in particular for *S*. *haematobium* infections (ranging from 21.4% to 71.4%) [29]. The combinations of two or more serologic tests, markedly increased the sensitivity of serology to almost 80% [29,30].

Other diagnostic techniques have been implemented more recently [24,31,32]. A Western Blot (WB) containing *S*. *haematobium* and *S*. *mansoni* soluble antigens was used on a limited number of subjects exposed to the recent outbreak of urinary schistosomiasis in Corsica, where a hybrid form between human *S*. *haematobium* and animal *S*. *bovis* was identified [18,33]. However, the accuracy of this test has never been formally evaluated. Previously, an immunoblot produced by the same manufacturer, using *S*. *mansoni* antigens only, had showed for this species a good accuracy (sensitivity 89.5% and specificity 100%) [34,35].

The urine Circulating Cathodic Antigen (CCA) is a rapid test, based on the identification in the urine of the genus-specific proteoglycan antigen of the schistosomal gut epithelium, regurgitated by the live adult worm only [36]. The CCA test has some limitations in identifying *S*. *haematobium* infection. In a Cochrane meta-analysis of studies conducted in endemic areas, the CCA test, compared to microscopy, showed an average sensitivity and specificity of 39% and 78%, respectively, for *S*. *haematobium* infection, and of 89% and 55%, respectively, for *S*. *mansoni*. This test has been utilized only recently with immigrants in Europe [37,38]. A rapid diagnostic test (RDT) incorporating antigens of adult *S*. *mansoni* cercarial transformation fluid for detection of antibodies in blood showed a sensitivity and specificity of 100% and 39.5%, respectively, in a schistosome-endemic area [39]. However, the reference standard used in that study was a fecal examination using Kato-Katz concentration technique, admittedly less sensitive than the index test. No studies have been carried out in non-endemic countries.

The main purpose of our study was to identify the more accurate screening strategy for schistosomiasis in a non endemic area.

The target condition was schistosomiasis (caused by *S*. *mansoni*, *S*. *haematobium*, or both) rather than the infection by either species. The rationale is that, with the exception of microscopy (and potentially PCR, that was not targeted by this study), the other techniques are not able to discriminate between the two species. Furthermore, the treatment of both infections is with the same drug praziquantel.

The main objective was to assess the accuracy of a series of diagnostic tests for the screening of schistosomiasis, including: a) the new, commercially available RDT (Schistosoma ICT IgG-IgM), and the Western Blot (Schisto II Western Blot IgG) (LD-BIO Diagnostics, Lyon France), both aimed at detecting *S*. *mansoni* and *S*. *haematobium* antibodies; the former test has been made commercially available recently (October 2015) and is based on the principle of the homogeneous sandwich (immunological reaction of 2 identical antigenic epitopes with the two binding sites of a bivalent antibody). b) the tests routinely used at CTD, namely: the circulating cathodic antigen (CCA) urine dipstick test for *S*. *mansoni* (NADAL CCA Bilharzia test, nal von minden, Germany), an enzyme-linked immunosorbent assay (ELISA Bordier Affinity Products, Crissier, Switzerland), and the microscopic examination of stool and urine.

## Materials and methods

### Ethics statement

Ethical clearance from Comitato Etico Provinciale di Verona e Rovigo: protocol n 33909 of July 13^th^, 2016. At the Centre for Tropical Diseases, all subjects submitted to any serological exam are asked to sign an informed consent for the anonymous storage of a serum specimen for any future research purpose. Parents’ or legal guardians’ consent is obtained for individuals of less than 18 years of age.

#### Study design

Retrospective, comparative diagnostic study, intended to estimate the sensitivity, specificity, positive and negative predictive values of the tests examined.

#### Study population

The study was carried out at the Centre for Tropical Diseases (CTD) of Sacro Cuore Hospital (Negrar—Verona, Italy), a referral center for tropical and parasitic diseases in the country. The study population was retrieved from the CTD patient database and was mostly composed by African recent asylum seekers that were routinely screened for schistosomiasis, regardless if symptomatic or not, in the context of a regional, pilot programme. The study was meant to include all consecutive subjects screened in the 2-year period considered (from March, 2014, when CCA urine dipstick was first made available). The study was carried out at the reference laboratory for parasitic diseases of CTD by experienced professionals.

The results of the routine screening tests (CCA, ELISA and microscopy) were retrieved from the patient database, while the new tests (ICT and WB) were performed for the study purpose on fully anonymized, coded serum samples, routinely stored and cryo-preserved at CTD. The archived specimens were kept frozen at -80°C from the day of the sample collection and the tests were executed within 24 hours of unfreezing. The study covered the two-year period from 1^st^ March, 2014 to 28^th^ February, 2016.

Inclusion criteria. Subjects of all ages submitted to routine screening for schistosomiasis with CCA urine dipstick, ELISA serology and microscopy of the stools and urine.

Exclusion criteria. a) Lack of informed consent for usage of biological samples for study purpose; b) Missing result for one of the routine screening tests (CCA urine dipstick, ELISA serology and microscopy,); c) Unavailability of ICT and/or WB result.

### Reference standard

The assessment of accuracy of all tests for schistosomiasis is hampered by the lack of a gold standard. In particular, microscopy (of stools and urine) is virtually 100% specific but lacks sensitivity [25–27]. On the contrary, serologic tests are known to be more sensitive but may provide false-positive results [24,29,30].

Therefore, the test accuracy was evaluated using three different standards:

1. Primary reference standard based on microscopy (see below for details)
2. Composite reference standard (CRS) (see below for details) [40,41].
3. Latent Class Analysis (LCA) (see below for details) [42].

#### Primary endpoints

Sensitivity, specificity, odds ratio, positive and negative predictive values of the index tests according to the primary reference standard.

#### Secondary endpoints

a) Sensitivity, specificity, odds ratio, positive and negative predictive values of the index tests according to CRS and LCA, respectively; b) Concordance between CRS and LCA in identifying true positive and true negative cases.

#### Test methods

Primary Reference standard: direct detection of *S*. *mansoni* or *S*. *haematobium* eggs in stools or urine. Composite Reference Standard. The subjects were classified as: **Infected** (denominator for sensitivity): positive microscopy OR (negative microscopy AND at least 2 concordant positives of the four index tests). **Not infected** (denominator for specificity): negative microscopy AND < 2 positives of the four index tests.

Index tests included four commercial tests: Urine CCA dipstick test (NADAL CCA Bilharzia test, Germany), Bordier ELISA (Bordier Affinity Products, Crissier, Switzerland), Schisto II Western Blot IgG (LD-BIO Diagnostics, Lyon, France) and Schistosoma ICT IgG-IgM (LD-BIO Diagnostics, Lyon, France).

A brief description of all the methods follows.

### Parasitological methods

From one to three urine samples were obtained (from 10 a.m. to 12 a.m) over consecutive days for each patient. The urine were subjected to CCA dipstick test and to filtration method for *S*. *haematobium* egg count on the day of the sample collection [36,43].

#### Urine CCA dipstick test

One drop of urine sample was added to the CCA urine cassette, following the manufacturer's instructions. Once absorbed, one drop of buffer was added and the result was read 20 minutes after. Valid tests were scored as positive or negative depending on whether or not a colored band appeared. The reading was done independently by two observers, and, in case of discordance, by a third observer. In case a result was judged equivocal by two observers (usually due to a very faint band), it was reported as indeterminate.

#### Urine microscopy

The remaining urine was shaken and filtered through a 25-mm diameter small meshed filter (12 μm Nucleopore), which was then placed on a labeled slide and examined under a microscope (100x) for Schistosoma eggs.

#### Stool microscopy

Three stool samples per patient were collected in formol on consecutive days. Each sample was submitted to formol-ether concentration and examined (100x) [26].

### Blood tests

Up to 10 ml of venous blood was collected from each patient in silica-coated tubes without anticoagulant. The serum was separated by centrifugation at 3000 rpm for 5 min. Bordier ELISA was executed on 10 μl of serum, and remaining aliquots were stored at -80°C. Both Schisto II Western Blot IgG and Schistosoma ICT IgG-IgM were performed using the latter serum. Cutoffs for each test were pre-determined prior to testing.

#### Bordier ELISA

The test detects Schistosoma IgG antibodies by using antigens from adult of *S*. *mansoni*. Briefly, 10 μl of serum were diluted with 2 ml of buffer before adding 100 μl to each ELISA microwell. According to the manufacturer's instructions, the result is positive when the absorbance of the analyzed sample is higher than the absorbance of the weak positive control (provided with the kit). In order to be able to compare results from different runs, we defined as positive samples those with: optical density (OD) of study sample/OD of weak positive serum ≥ 1 (normalized OD). The test was carried out in double and, in case of discordant results, it was further repeated on another blood sample, in order to avoid indeterminate results.

#### Schisto II Western Blot IgG

The test was carried out according to the manufacturer's instructions. Briefly, the *S*. *mansoni* + *S*. *haematobium* antigen strips (supplied with the kit) were incubated with 25 μl of sera diluted in Tris-NaCl sample buffer for 90 minutes. Then, a first washing step with Tris-NaCl washing buffer permitted to remove any unbound sample components. Later, the strips were incubated with an alkaline phosphates—anti human IgG conjugate for 60 minutes. After a second washing step, the whole complex was revealed by the corresponding substrate-chromogenic solution containing nitroblue tetrazolium and 5-bromo-4-chloro-3-indolylphosphate as a dark blue-purple color. The color development was stopped after 60 minutes by washing the strips with distilled water. The strips were dried and glued to paper for reading. The Schistosoma-specific IgG, if present in the sample, appeared as violet colored bands. Valid tests were read as positive depending on the presence of one of the following: 1) band P22-24; 2) a large band P30-34; 3) a thin band P30-34 plus at least one among bands P8, PP9, P10, P12-13, P14-15, P18. The strips were read independently by two observers, and, in case of discordance, by a third observer. In case a result was judged equivocal by two observers (typically for the appearance of a “shadow” rather than a clear band), it was reported as indeterminate.

#### Schistosoma ICT IgG-IgM

This immune-chromatographic test (ICT) was carried out according to the manufacturer's instructions. Briefly, 30 μl of serum were added to a cassette, followed by 2 drops of the eluent (supplied with the kit). The result was read after 20 minutes. The tests were read as positive or negative depending on whether or not a colored band had appeared. The reading and the interpretation were carried out the same way as for CCA (see above).

#### Cost

The approximate cost per test (in Italy, and without considering the labour costs) is € 7 for ELISA, € 9 for CCA, € 14 for ICT and € 33 for WB.

#### Handling of indeterminate results

For the analysis, all indeterminate results were classified as positive. All reference and index tests were executed and read by highly trained, senior staff of the CTD laboratory. Four senior staff members performed and read all the tests, according to the procedures outlined above.

#### Blinding

Microscopy, serology and urine CCA dipstick test were carried out at CTD by different laboratory personnel. The staff performing the tests carried out on the frozen specimens (ICT and WB) had no access to the source codes and therefore were blinded as of the results of the previous tests, as well as of the results of either test.

#### Statistical analysis

For both the primary and secondary endpoints, the sensitivity of each index test was calculated as the proportion of positive results over all infected subjects according to the primary and composite reference standards, respectively. Uncertainty was quantified using the 95% confidence intervals. Similarly, the specificity was calculated as the proportion of negative results over all not infected subjects according to the primary and composite reference standard, respectively. Uncertainty was quantified the same way as above.

A further analysis was performed using the method of Latent Class Analysis (LCA).

Latent class models (LCMs) combine the results of multiple diagnostic tests through a probabilistic model to obtain estimates of disease prevalence and diagnostic test accuracy in situations where there is no single, accurate reference standard [42].

Diagnostic studies that apply LCMs treat the target disease status as an unmeasured (”latent”) categorical variable with K classes, reflecting the levels of the underlying disease. The manifest variables, the outcomes of T (binary) diagnostic tests, are considered to be imperfect classifiers of the disease status. The LCM describes a statistical model relating the manifest variables to the latent disease status.

When K = 2, it is assumed that the two latent classes correspond to a class of subjects in which the target disease is present and a class of subjects in which the target disease is absent. Parameter estimates obtained from the 2-class LCM are interpreted as estimates of the sensitivity and specificity of each test and the prevalence of the target disease (i.e., the prior probability that the target disease is present).

We assumed the conditional independence among the tests.

The analysis was performed by using LCA, random LCA and extended LCA R’s libraries.

Predictive values (PPV, NPV) were then estimated, based on the prevalence of the infected subjects obtained with the three methods (microscopy, CRS and LCA).

We also assessed the concordance between CRS and LCA in classifying the study subjects. Considering as cases all subjects classified in Class 1 by LCA, and negatives (non-cases) all subjects classified in Class 2 by LCA, we compared the proportion of subjects classified as cases and non-cases by CRS and LCA, respectively.

Cohen’s Kappa measure (with its 95% confidence interval) was used to assess the agreement as follows: K ≤ 0, no agreement; K = 0–0.20, poor agreement; K = 0.21–0.40, fair agreement; K = 0.41–0.60, moderate agreement; K = 0.61–0.80, substantial agreement; and K = 0.81–1.00, nearly perfect agreement [44,45].

#### Ethical issues

Samples were anonymously coded, unlinked from any information identifying the source individuals. At the Centre for Tropical Diseases, all subjects submitted to any serologic exam are asked to sign an informed consent for the anonymous storage of a serum specimen for any future research purpose. Parents’ or legal guardians’ consent is obtained in case of individuals of less than 18 years of age. Specimens lacking the consent are immediately discarded. Although the study was retrospective and no action on patients was involved, the study protocol was nevertheless submitted to the Ethics Committee of the Coordinating Site (Comitato Etico Provinciale di Verona e Rovigo) for approval. The latter acknowledged the study protocol and formally authorized the study (protocol n. 33909 of July 13^th^, 2016).

## Results

**Fig 1:**
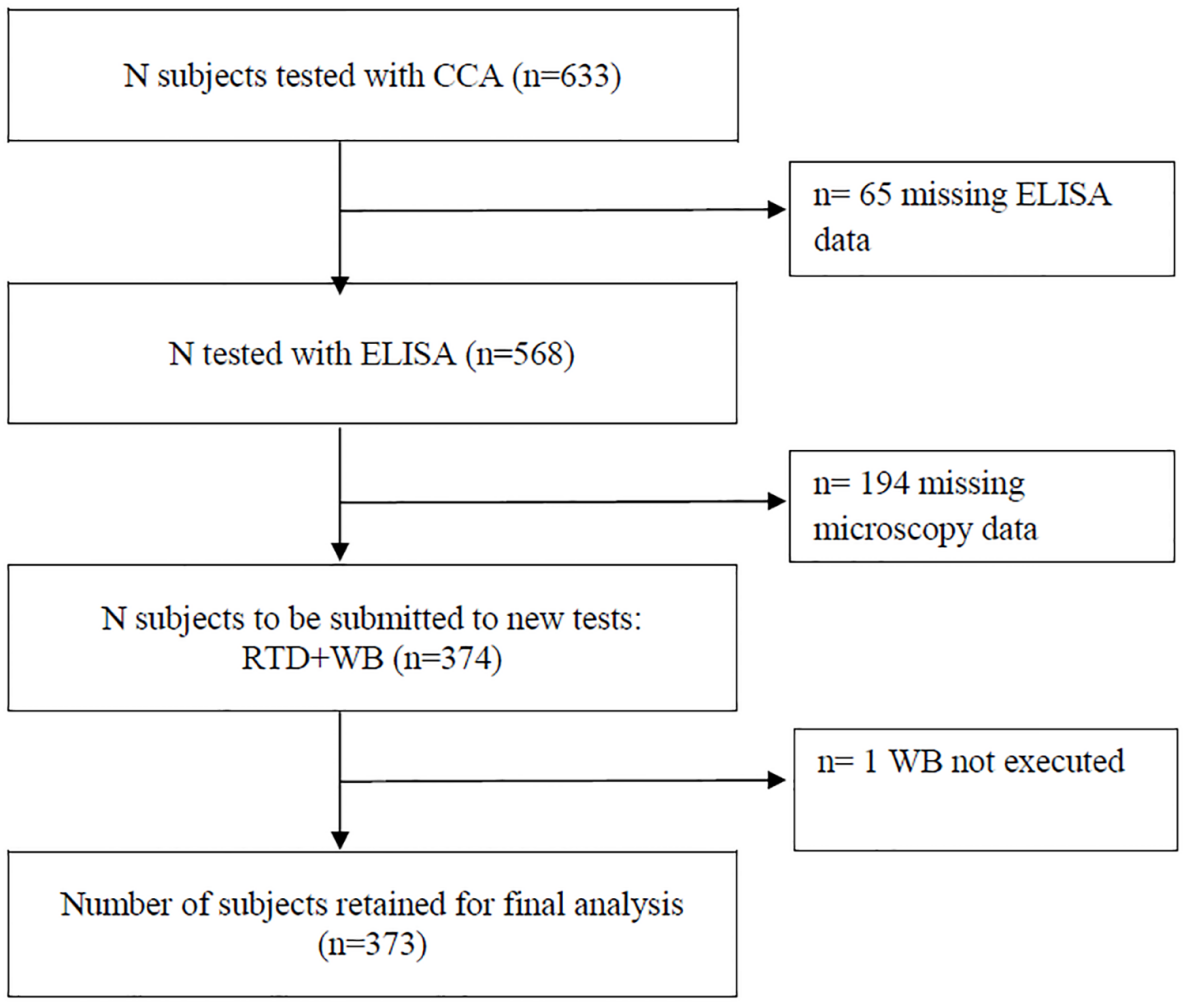
Study flow chart.

The flow of participants is described in Fig 1. The study subjects were predominatly male (323/373 or 87%). The median age was 25 years (IQ range, 20–31). Seven subjects were minors, the youngest one was a 11-year-old boy.

### Results according to the primary reference standard

The proportion of positive microscopic results in the population under study was 65/373 (17.4%). In particular, *S*. *mansoni* eggs were found in the stools of 32/373 subjects (8.6%) and *S*. *haematobium* eggs in the urine of 40/373 subjects (10.7%). Seven subjects (1.9%) had both the infections.

The results of the 4 index tests according to microscopy are reported in Table 1. Patients were classified as microscopically positive if ova of at least one of the two species were identified in the stools or urine. ICT and WB presented the highest sensitivity (94% and 92%, respectively) and negative predictive values (NPV): 98%, both tests. CCA demonstrated a low sensitivity (48%), while its specificity was the highest (93%).

**Table 1:** Accuracy and predictive values using microscopy as the gold standard (prevalence 65/373 = 17.4%).

Test	Sensitivity	Specificity	PPV	NPV
CCA	31/65^1^ (48%, CI 40–65)	285/308^2^ (93%, CI 89–95)	57%	89%
ELISA	53/65 (82%, CI 70–90)	258/308 (84%, CI 79–88)	51%	96%
WB	60^3^/65 (92%, CI 83–98)	222/308^4^ (72%, CI 67–77)	41%	98%
ICT	61^5^/65 (94%, CI 85–98)	191/308^6^ (62%, CI 56–68)	34%	98%

^1^ Including 1 borderline result classified as positive as explained in Methods

^2^ Including 6 borderline results classified as positive as explained in Methods

^3^ Including 1 borderline result classified as positive as explained in Methods

^4^ Including 1 borderline result classified as positive as explained in Methods

^5^ Including 5 borderline results classified as positive as explained in Methods

^6^ Including 23 borderline results classified as positive as explained in Methods

Considering *S*. *mansoni* only, CCA sensitivity increased to 72% (23/32), while that of the other tests resulted similar, in particular: ELISA, 84% (27/32); WB, 91% (23/32); and ICT, 94% (29/32).

Considering samples with positive microscopy for *S*. *haematobium* and negative for *S*. *mansoni*, CCA sensitivity dropped to 24% (8/33), while that of the other tests, again, resulted similar, in particular: ELISA, 79% (26/33); WB, 94% (31/333); and ICT, 94% (31/33).

We also assessed if the presence of haematuria significantly influenced the proportion of CCA positivity. This data was available for about half of the study subject (185/373 or 49.6%). Considering subjects who resulted microscopically negative to both species, CCA resulted positive in 6/43 subjects with haematuria (14%, CI 5–28) and in 9/73 subjects without haematuria (11%, CI 5–20).

### Results according to the composite reference standard (CRS) and to Latent Class Analysis (LCA) (Table 2)

**Table 2:** Accuracy and predictive values according to CRS and to LCA.

Test	Sensitivity (95% CI)		*Specificity (95% CI)*		*PPV*		*NPV*	
	CRS	LCA	CRS	*LCA*	*CRS*	*LCA*	*CRS*	*LCA*
**CCA**	29% (22–37)	29% (21–37)	95% (91–97)	*93% (89–96)*	*78%*	*76%*	*68%*	*70%*
**ELISA**	71% (63–78)	76% (67–84)	99.6% (98–100)	*99*.*4% (95–100)*	*99%*	*99%*	*84%*	*88%*
**WB**	92% (86–96)	94% (88–97)	94% (90–97)	*91% (85–95)*	*90%*	*85%*	*95%*	*97%*
**ICT**	96% (91–99)	96% (90–99)	83% (77–87)	*79% (73–84)*	*78%*	*72%*	*97%*	***97%***
**Micro**	45% (37–54)	48% (39–57)	100%^1^	*99% (96–100)*	*100%*	*98%*	*74%*	*77%*

^1^ by definition

The proportion of positive results according to the CRS was 38.6% (144/373). According to LCA modeling, the proportion of subjects classified in Class 1 was 35.6%. The results are detailed in Table 2.

ICT confirmed the highest sensitivity (96% for both models), followed by WB. CCA was again the test with the lowest sensitivity, that resulted very low (29%, both models). On the other hand, although the specificity of CCA was confirmed to be higher than 90%, both CRF and LCA identified ELISA as the indirect test with the highest specificity, being over 99%.

**Fig 2:**
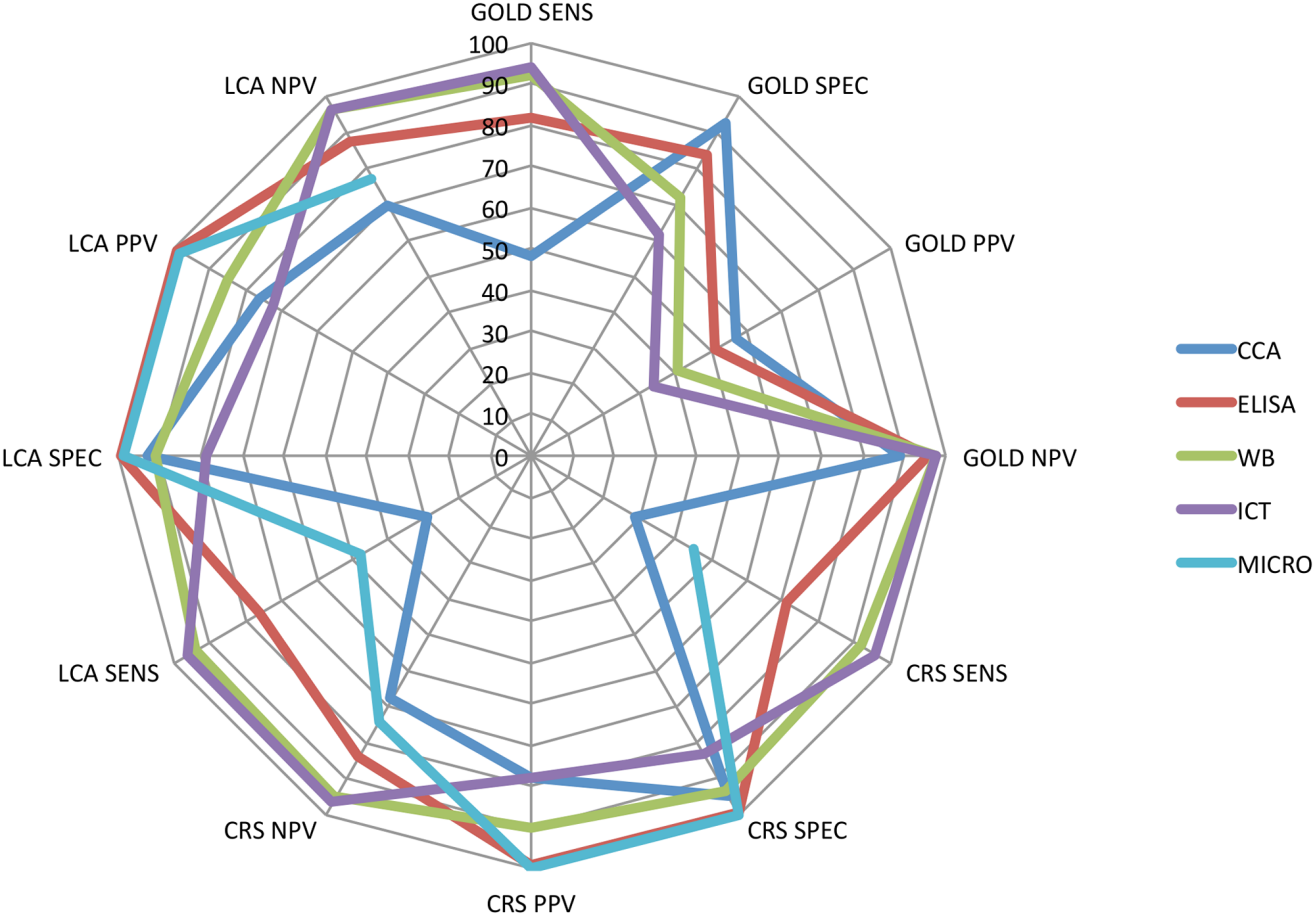
Graphical representation of the main results (sensitivity, specificity, positive predictive value, negative predictive value) of the 5 tests examined, according to the three different reference standards used. GOLD = primary reference standard, based on microscopy; CRS = composite reference standard; LCA = Latent Class Analysis.

The accuracy and predictive values, according to the three reference standards used, is summarized in Fig 2.

### Concordance of the subject classification between CRS and LCA models

Subjects with at least 2 concordant positive results of the index tests OR with a positive microscopy (irrespective of the other results) had 91.1% probability of being classified as cases by LCA, while all the others (negative microscopy and <2 positive index tests) had 99.5% probability of being classified as non-cases by LCA.

The concordance between the two methods of classification was excellent (Cohen’s kappa = 0.92, indicating near-perfect agreement).

### Predictive values of a combination of 2 tests (ICT plus a second index test), according to LCA

Assuming using ICT as a screening test (then with a second, confirmatory test in case of ICT positivity), we assessed, using the LCA model, the positive and negative predictive values of a combination of a positive ICT (the test with the highest sensitivity) with a positive or a negative second test, respectively. The results are reported in Table 3.

**Table 3:** Predictive values of a combination of positive ICT and a positive (PPV) or a negative (NPV) second test, according to Latent Class Analysis (LCA).

Second test	PPV	NPV
**CCA**	90%	47%
**ELISA**	98%	51%
**WB**	82%	87%
**Microscopy**	100%	43%

## Discussion

### Main results

Schistosomiasis prevalence was very important in the study population, composed for the vast majority of asylum seekers. Even when considering microscopy only, that is not sufficiently sensitive, the proportion of positives was higher than 17%. According to CRS and LCA, this proportion was very similar, 38.6% and 35.6%, respectively.

The urinary, rapid antigen test CCA was unsatisfactory: its sensitivity was poor, and this, with any reference standard used, makes it inadequate for screening purpose. The higher sensitivity found by other studies [37] probably reflects higher, average parasitic loads and a different reference standard based on microscopy only. Therefore, CCA is possibly a useful test (for *S*. *mansoni*) when the goal is to identify infections in an endemic country, but, according to our results, its sensitivity in non-endemic settings should be better assessed, before considering it as a potential screening tool, considering that the goal is to find and treat any infection, including chronic infections with a low egg output.

WB showed an excellent combination of sensitivity and specificity, although the latter was not as high as one might expect by this technique. According to the manufacturer, a single band is sufficient to define a positive result. It is possible that a more conservative definition (i.e. requiring at least two positive bands) would bring the test specificity closer to 100%, though, expectedly, at the expense of some loss of sensitivity. We plan to investigate this with the next, longitudinal study (see below).

ELISA was the test providing the most surprising results, as it was found to be virtually 100% specific according to both CRS and LCA. This result was not expected, as serologic tests in general are held to give a variable proportion of false positive results, due to cross-reaction with other pathogens and to other reasons [24]. We tried to figure out if a possible selection bias could explain this unexpected result, but we couldn’t find any, although the retrospective design of the study suggests that the results should be interpreted with some caution.

According to both CRS and LCA, the sensitivity of microscopy was poor, thus confirming that finding schistosome eggs in the stools or urine is diagnostic, but their absence does not by any mean rule out the infection.

### Concordance between the composite reference standard and Latent Class Analysis

All the main results, both of the infection prevalence in the study population and of the accuracy of the different tests, were very similar between the two models, indicating a satisfactory robustness of the (independent) methods used. The agreement between the two models in classifying the study subjects was nearly perfect (Cohen’s kappa test = 0.92).

### How to plan a screening program for new immigrants and refugees

Should a screening program be planned, it should rely on a sensitive approach, that minimizes the risk of leaving infected subjects without the necessary treatment.

Regardless the reference standard used, the ICT resulted the most sensitive test, with the potential of being used as a single screening test (NPV >97% according to the primary and composite reference standard, and to LCA). The PPV of ICT was low when using the primary reference standard (reflecting the low specificity when a poorly sensitive test is used as reference), but reasonably high according to both the composite reference standard and the LCA, thus probably justifying a treatment, in a screening context, even without a confirmation test.

Would a second test necessary/useful? The answer to this question depend on the context. Newly arrived immigrants/asylum seekers are often a very mobile group, and it is crucial to be able to screen (and treat the positives) at the first visit. The ICT appears to be sufficiently accurate for this purpose. On the other hand, in a clinical context, a confirmatory test would be obviously indicated. A positive microscopy would be the only test providing the certainty of infection, but a negative result would by no mean rule out the infection (Table 3). ELISA is a good confirmer but a weak excluder, too. (Table 3). Only the combination of more, concordantly negative tests would safely exclude the infection in a subject with a positive ICT. In summary, ICT is the ideal test for screening purpose. It is a simple test, not even requiring a laboratory or any special equipment, can be read in a few minutes and is the only test that virtually rules out the infection, if negative. This test would be even more suitable for screening purpose if validated for use on whole blood obtained through a finger prick. Unfortunately the producer has not validated this procedure.

In a screening context, this test can be used alone as a tool to decide the treatment, the only reasonable alternative being, in a high prevalence population such as our study population, a presumptive treatment of all immigrants coming from high prevalence countries, considering the potential severity of the chronic infection and the relatively harmless and highly effective treatment available (praziquantel). The cost-effectiveness of either approach was beyond the scope of this research and should be planned as a further study.

#### Strengths and weaknesses

We believe that a major strength of our study is the reference standard used. Direct demonstration of eggs in stools and/or urine is of course diagnostic, but this technique is also known to lack sensitivity, as infections with a low parasitic load are easily missed [25–27]. It is clear that any test with a poor sensitivity cannot be used as a gold standard to evaluate the accuracy of alternative, index tests, as discordant results would be impossible to classify. Yet, most diagnostic studies in parasitology, and on schistosomiasis in particular, rely on microscopy as the gold standard [27], and only rarely alternative reference standards are used, although the latter are the only acceptable methods when a gold standard is missing [40–42]. In this study we used, besides microscopy, two of the alternative methods that are recommended when a gold standard is not available, that are, a CRS and the LCA. Both methods provided very similar results, for all the index tests considered, while when microscopy was used as reference, sensitivity of the index tests was similar as with the alternative methods, but specificity was invariably lower (reflecting the likely misclassification of discordant results).

We acknowledge also some limitations of the models used. As far as the CRS is concerned, it may be argued that it is not correct to include an index test among the tests composing the reference standard. Moreover, the latter tests should not be conditionally dependent, while in fact three of them were based on the same principle, that is, antibody detection, and therefore conditional independence cannot be assumed. Besides, antibody detection does not necessarily reflect an active infection.

In our retrospective, real-life study, we aimed at estimating the accuracy of all available tests, and this is the reason why all the index tests were also part of the reference standard. This was the only way of obtaining the same denominator of infected/not infected for all the tests examined. Precisely because we were aware of the limitations of the CRF used, we decided to also use a third, alternative method, namely the LCA.

More in general, the main weakness of our study was the retrospective design. Although most of the tests were carried out for screening purpose and not because of clinical suspicion, some selection bias cannot be ruled out. Moreover not all the tests were always available during the study period, and the subjects lacking any of the tests under study had to be excluded from the analysis. Finally, the estimation of the prevalence in a hospital setting may not be representative of the general population, although in this case, as explained above, most tests were carried out on subjects submitted to a pilot screening, regardless the presence/absence of symptoms.

Both ICT and CCA tests imply the visual reading by the observer of a band appearing within 15 minutes on the device. Theoretically, a classification of positive results (e.g. “strong positives” and “weak positives”) would be possible, based on the band intensity, also considering that the few results originally classified as undetermined obviously concerned faint positives for which an agreement was not reached). Unfortunately the test results were only classified as positive, negative or undetermined, therefore this distinction was not possible and will be certainly taken into account in a prospective study (see below). Automatic readers could possibly improve the use of these and other qualitative diagnostic tools in the future.

#### Future research

We are planning an extensive, multi centre, longitudinal diagnostic study in order to assess the best diagnostic and screening strategy for schistosomiasis. Bio-molecular methods (that were not included in this study) will be also assessed. We are also planning to compare the results of the ICT performed after finger prick with those on serum obtained according to the standard procedure. If they are concordant, the use of ICT will be greatly facilitated outside health facilities.

### Conclusion

The new, rapid diagnostic test ICT is a suitable screening tool for schistosomiasis. A positive result should ideally be confirmed by a second test. ELISA and of course microscopy are the best confirmers of a positive ICT. Nevertheless, neither second test, if negative, is sufficient to rule out the infection. In a screening context, the ICT should probably used alone as a decision tool about treating or not the infection, the only practical alternative being a presumptive treatment of the population at risk.

## Supporting information

S1 File Study database. (XLSX)
